# Comparative Clinical Outcomes of Different Orbitotomy Approaches in the Surgical Management of Orbital Tumors

**DOI:** 10.1155/joph/9362866

**Published:** 2026-04-13

**Authors:** Karen Sevterteryan, Kamo Shaboyan, Gagik Hakobyan

**Affiliations:** ^1^ Department of Oral and Maxillofacial Surgery, Yerevan State Medical University After M. Heratsi, Yerevan, Armenia, ysmu.am; ^2^ Department of Military Surgery, Yerevan State Medical University After M. Heratsi, Yerevan, Armenia, ysmu.am; ^3^ Department of Dental Research Cell, Dr. D. Y. Patil Dental College and Hospital, Dr. D. Y. Patil Vidyapeeth, Pune, India, dpu.edu.in

**Keywords:** endoscopic orbitotomy, orbital surgery, orbital tumor, orbitotomy

## Abstract

**Background:**

Optimal surgical management of orbital tumors requires precise selection of the surgical approach to achieve complete tumor removal while preserving visual function and maintaining cosmetic outcomes.

**Objective:**

This study aimed to evaluate and compare the clinical outcomes of different orbitotomy approaches—including lateral, inferior, supraorbital, and endoscopic endonasal techniques—in the surgical management of orbital tumors.

**Methods:**

A retrospective single‐center study was conducted on 68 patients (36 men and 32 women; mean age: 42.1 ± 14.3 years) who underwent surgical treatment for orbital tumors between 2018 and 2024. Preoperative evaluation included comprehensive ophthalmologic examination, CT and MRI imaging, and histopathological confirmation. The choice of surgical approach was guided by tumor location: lateral orbitotomy (*n* = 17), inferior orbitotomy (*n* = 21), supraorbital orbitotomy (*n* = 14), and endoscopic endonasal approach (*n* = 16). Continuous variables are presented as mean ± SD. Paired *t*‐tests and *χ* ^2^ tests were used for statistical analysis, with *p* < 0.05 considered statistically significant.

**Results:**

Visual acuity and ocular motility were preserved in 65 of 68 patients (95.6%). Mean postoperative orbital volume at six months was 26.31 ± 1.28 mL on the operated side and 25.71 ± 1.82 mL on the contralateral side, with no significant difference (*p* > 0.05). Globe projection differences were < 2 mm in 94.1% of patients. Postoperative complications were minimal, with only a few cases of transient diplopia.

**Conclusion:**

Selection of orbitotomy approach based on tumor localization allows safe and effective surgical management of orbital tumors. Both traditional and minimally invasive approaches can provide favorable functional and cosmetic outcomes when applied according to anatomical indications.

## 1. Introduction

Primary orbital tumors encompass a heterogeneous group of neoplastic, inflammatory, and infectious lesions [1]. Approximately 80% of orbital tumors are benign, whereas 20% demonstrate malignant behavior. Among benign lesions, dermoid cysts and cavernous hemangiomas represent some of the most frequently encountered pathologies [2–4]. Despite their predominantly benign nature, orbital tumors may cause significant functional impairment due to the confined anatomical space of the orbit.

Lesions with high malignant potential may present more aggressively and require urgent evaluation. Comprehensive diagnostic assessment, including advanced imaging modalities and histopathological verification, is essential for accurate diagnosis and surgical planning. To minimize surgical risk and postoperative morbidity, new surgical trends favor minimally invasive techniques whenever possible [5, 6].

The most common symptoms of orbital tumors include pain in the eyeball region, displacement and limitation of ocular motility, diplopia, visual acuity impairment, and ocular mucosal edema [7], as well as enophthalmos and hypophthalmos [8]. Tumors with high malignant potential are exceptions and require a comprehensive set of diagnostic methods when suspected [9].

The zygomaticomaxillary complex (ZMC), located in the midface, plays an important functional and aesthetic role, which creates significant challenges for surgical reconstruction and correction of deformities in this area [10, 11]. Its intricate anatomy and proximity to vital neurovascular structures present substantial challenges for surgical access, tumor resection, and reconstruction.

Therefore, selection of the surgical approach is primarily determined by tumor location relative to the optic nerve, extraocular muscles, and orbital walls. Traditional extracranial orbitotomy approaches include lateral, transethmoidal, transconjunctival, transmaxillary, and supraorbital techniques. More recently, minimally invasive endoscopic endonasal approaches—such as medial‐inferior extraconal, transmaxillary extraconal, and medial intraconal access—have expanded the surgical armamentarium. Advances in computer‐assisted design, three‐dimensional planning, and intraoperative navigation have further enhanced surgical precision and safety in orbital and craniofacial procedures.

The choice of surgical approach for removing tumors from the orbit is dictated by the localization of the tumor in relation to the orbit [11–13]. Computer‐aided design and manufacturing technologies, combined with intraoperative navigation, are now widely used in various craniofacial surgeries. Preoperative planning and intraoperative surgical navigation can provide additional precision and safety during fundus reconstruction, improving clinical outcomes [14, 15].

Management of orbital tumors requires a multidisciplinary team involving ophthalmologists, neurosurgeons, otolaryngologists, radiologists, and pathologists to optimize diagnostic accuracy and therapeutic outcomes. An interprofessional team is essential for the diagnosis and treatment of patients with benign orbital tumors [16].

Extracranial orbitotomy approaches include [17]

• Lateral orbitotomy.
• Transethmoidal.
• Transconjunctival.
• Transconjunctival.
• Transmaxillary.
• Supraorbital.

Endoscopic endonasal approaches include

• Medial‐inferior extraconal approach.
• Transmaxillary extraconal approach.
• Medial intraconal approach.

Currently, lateral orbitotomy is performed by a multidisciplinary team consisting of a neurosurgeon and an ophthalmologist to plan and implement safe and effective treatment.

The present study aims to evaluate and compare the clinical effectiveness and surgical outcomes of different orbitotomy approaches—including lateral, inferior, supraorbital, and endoscopic endonasal techniques—in the management of orbital tumors.

## 2. Materials and Methods

### 2.1. Study Design and Patients

This retrospective study included 68 patients (36 men and 32 women; mean age: 42.1 years; range: 29–75 years) who underwent surgical removal of orbital tumors. All patients were treated at a tertiary referral center by a multidisciplinary team.

Inclusion criteria were

1. Patients diagnosed with orbital tumors requiring surgical removal.
2. Availability of preoperative computed tomography (CT) imaging or magnetic resonance imaging (MRI).
3. Minimum postoperative follow‐up of 12 months.

Exclusion criteria included

1. Incomplete clinical or imaging data.
2. Patients treated with nonsurgical therapy.
3. Tumors with extensive intracranial extension requiring neurosurgical cranial approaches.

### 2.2. Preoperative Evaluation

All patients underwent comprehensive clinical assessment, including detailed medical history, evaluation of presenting complaints, and general physical examination. Ophthalmological examination included visual acuity testing (visometry), perimetry, campimetry, ophthalmometry, and exophthalmometry. Standardized preoperative photographic documentation was performed.

Routine laboratory investigations included complete blood count and urinalysis. Preoperative consultations with a therapist and anesthesiologist were obtained in all cases.

### 2.3. Imaging and Diagnostic Workup

To establish diagnosis and plan the surgical approach, the following investigations were performed:

• Exophthalmometry.
• Orbital ultrasonography with Doppler assessment.
• CT.
• MRI.
• Biopsy with histopathological examination.

### 2.4. Tumor Characteristics

Tumor size and relationship to surrounding anatomical structures were assessed using CT and MRI imaging, as these parameters are critical determinants in selecting the appropriate orbitotomy approach. Tumor localization was classified according to orbital compartment (lateral, medial, superior, and inferior) and relative position to the optic nerve (intraconal or extraconal), confirming the heterogeneity of lesion distribution and supporting the need for a differential surgical approach.

Histopathological examination confirmed various tumor types: pleomorphic adenoma of the lacrimal gland (*n* = 11), cavernous hemangioma (*n* = 9), osteoma (*n* = 9), dermoid cyst (*n* = 8), lymphoma (*n* = 7), pseudotumor (*n* = 7), solitary fibroma (*n* = 6), inflammatory granuloma (*n* = 6), and lymphangioma (*n* = 5) (Table 1).

**TABLE 1:** Distribution of orbital tumor types and localization (*n* = 68).

Tumor type	Total (*n* = 68) (%)	Lateral	Medial	Superior	Inferior
Solitary fibroma	6 (8.8)	3	0	2	1
Dermoid cyst	8 (11.8)	2	1	3	2
Cavernous hemangioma	9 (13.2)	4	0	2	3
Lymphoma	7 (10.3)	3	2	0	2
Lymphangioma	5 (7.4)	2	1	2	0
Inflammatory granuloma	6 (8.8)	1	2	2	1
Osteoma	9 (13.2)	4	0	2	3
Pleomorphic adenoma (lacrimal gland)	11 (16.2)	5	2	3	1
Pseudotumor	7 (10.3)	2	3	1	1
**Total**	**68 (100)**	—	—	—	—

*Note:* Bold values represent the cumulative total of all tumor cases and confirm that the percentages are calculated based on the entire study cohort (*n* = 68).

### 2.5. Clinical Presentation

At admission, the most common symptom was proptosis (65/68; 95.6%), followed by decreased visual acuity (28/68; 41.2%), diplopia (12/68; 17.6%), headache (12/68; 17.6%), retrobulbar pain (9/68; 13.2%), and trigeminal hypesthesia (7/68; 10.3%).

### 2.6. Preoperative Planning and 3D Reconstruction

Tumor resection was digitally simulated using computer‐assisted planning. Preoperative CT data were uploaded into specialized software to reconstruct a three‐dimensional model of the orbit. A mirror image of the unaffected side was used to guide anatomical restoration and surgical planning.

### 2.7. Surgical Approach Distribution

**FIGURE 1:**
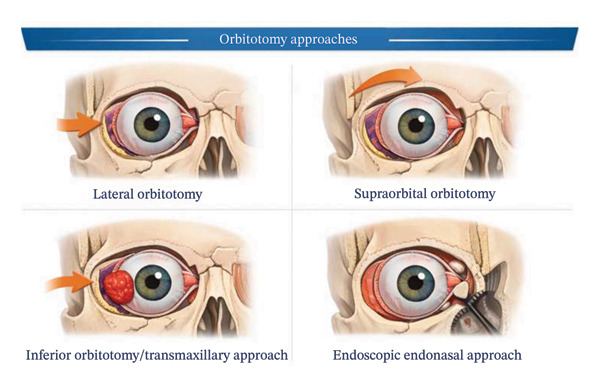
Surgical approach.

The surgical approaches were distributed as follows (Table 2 and Figure 1):

• Lateral orbitotomy: 17 patients (25.0%).
• Inferior orbitotomy: 21 patients (30.9%).
• Supraorbital orbitotomy: 14 patients (20.6%).
• Endoscopic endonasal approach: 16 patients (23.5%).

**TABLE 2:** Surgical approaches for orbital tumor removal (*n* = 68).

Surgical approach	Patients (*n*)	Percentage (%)
Lateral orbitotomy	17	25.0
Inferior orbitotomy	21	30.9
Supraorbital orbitotomy	14	20.6
Endoscopic endonasal approach	16	23.5
**Total**	**68**	**100**

*Note:* Bold values indicate the total number of patients and the corresponding percentage of the entire study cohort (*n* = 68).

### 2.8. Surgical Techniques

The choice of surgical approach was determined by tumor localization relative to the optic nerve and orbital walls (Figure 1).

• **Lateral orbitotomy** was performed for lesions located lateral to the optic nerve.
• **Medial orbitotomy**, including anterior medial micro‐orbitotomy, was used for tumors medial to the optic nerve.
• **Inferior orbitotomy** or **endoscopic endonasal approach** was selected for tumors located in the inferior orbit.

### 2.9. Inferior Orbitotomy Technique

For tumors involving the inferior posterior orbit, a transmaxillary approach was utilized. After exposure of the anterior wall of the maxillary sinus and orbital floor, a quadrangular bone window (3–3.5 × 1.5–1.8 cm) was created. The orbital floor segment was carefully mobilized to gain access to the retro‐orbital space while preserving the infraorbital nerve and orbital soft tissues. Following tumor removal, the bone segment was repositioned and secured with fixation sutures, restoring anatomical continuity.

This technique provides safe access to the inferior retro‐orbital space while preserving orbital integrity and minimizing the risk of enophthalmos.

### 2.10. Clinical Case

**FIGURE 2:**
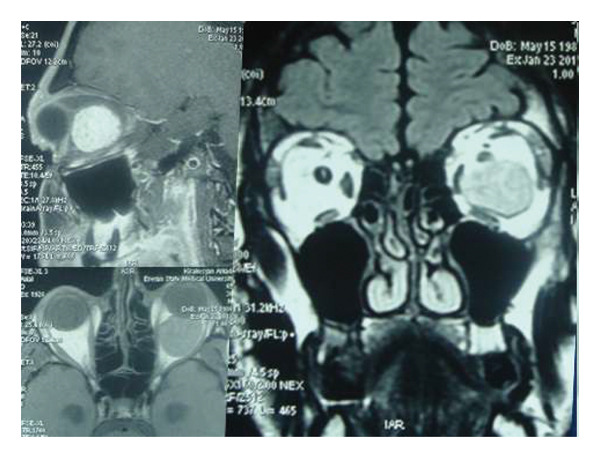
Preoperative MRI of the left orbit. Coronal, axial, and sagittal sections demonstrate a lesion in the left lateral orbit.

**FIGURE 3:**
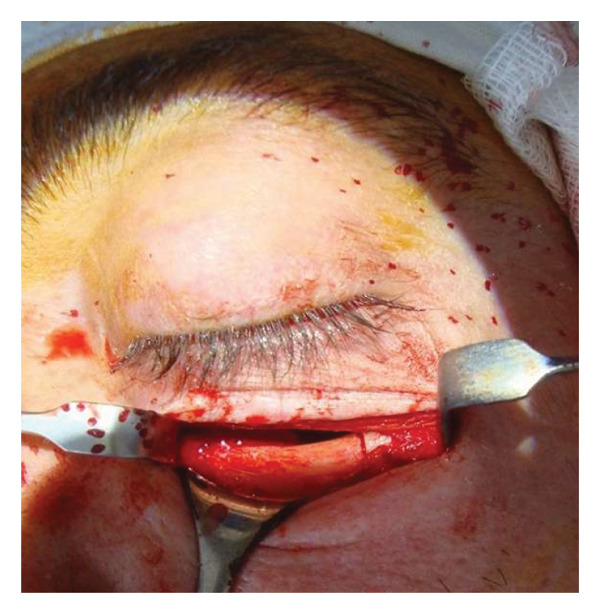
Intraoperative exposure of the lower orbital wall. The orbital floor is resected, and the bone fragment is carefully removed to access the retro‐orbital space.

**FIGURE 4:**
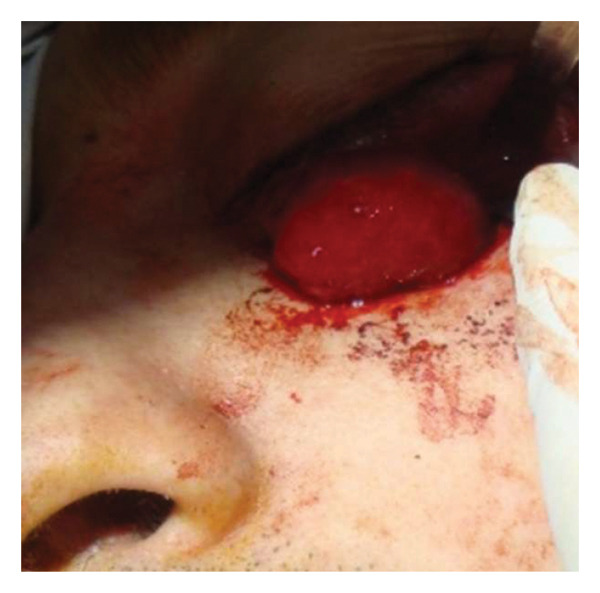
Cavernous hemangioma located in the intraconal space of the left lateral orbit, visualized intraoperatively prior to excision.

**FIGURE 5:**
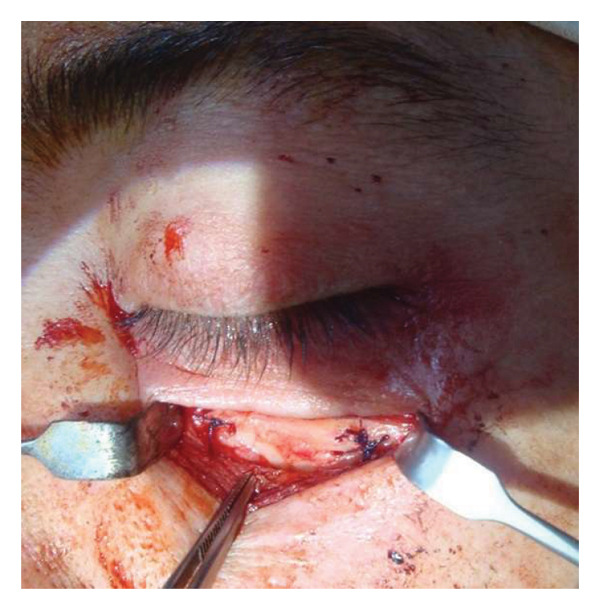
Post‐tumor removal. After complete excision of the cavernous hemangioma and achievement of hemostasis, the bone flap is repositioned and secured. The skin incision is then closed.

A 40‐year‐old patient (A.H.) presented with a 5‐year history of upper eyelid swelling. Diagnostic workup confirmed cavernous hemangioma of the intraconal space of the left orbit. Based on tumor location, the inferior orbitotomy technique was used (Figures 2, 3, 4, and 5). After tumor removal, the bone flap was returned to its anatomical position and secured, and the skin incision was closed with standard suturing.

### 2.11. Statistical Analysis

Comparisons between continuous variables were performed using the paired Student’s *t*‐test. Categorical variables were analyzed using the chi‐square (*χ* ^2^) test. A *p* value < 0.05 was considered statistically significant.

Statistical analysis was performed using SPSS software (IBM Corp., USA). Continuous variables were expressed as mean ± standard deviation (SD), and categorical variables were presented as frequencies and percentages. Differences between continuous variables were analyzed using the paired Student’s *t*‐test, while categorical variables were compared using the *χ* ^2^ test. A *p* value < 0.05 was considered statistically significant.

## 3. Results

All patients were followed up for 1 to 3 years. Visual acuity was assessed using the Snellen visual acuity scale, and ocular motility was evaluated through standard ophthalmologic examination. Orbital volume measurements were obtained from postoperative CT imaging, while cosmetic outcomes were assessed clinically based on globe symmetry and facial contour.

The postoperative projection of the eyeball and orbital volume on the reconstructed and unaffected sides were measured using CT and MRI images obtained six months after initial surgery. Orbital volume was calculated based on serial axial CT and MRI slices.

Following orbital floor reconstruction, quantitative analysis demonstrated preservation of visual acuity and ocular motility in 95.6% of patients (65/68), with minimal postoperative complications, including transient diplopia and mild restriction of ocular motility.

The results revealed no statistically significant differences in orbital volume or globe projection between the reconstructed and unaffected sides. Clinically significant enophthalmos was assessed based on differences in eyeball projection; a difference of less than 2 mm was considered clinically insignificant.

### 3.1. Orbital Volume and Globe Projection

**FIGURE 6:**
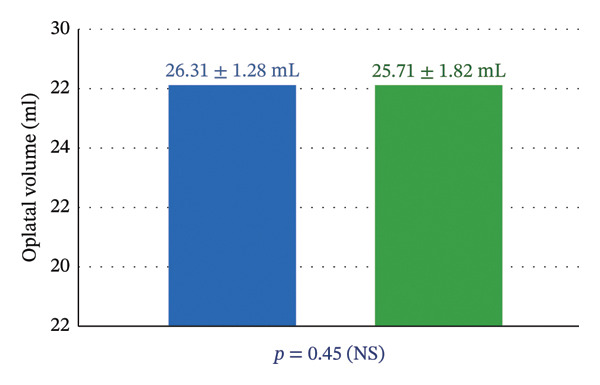
Orbital volume measurements (6 months postoperative).

Six months after surgery, orbital volume measurements were as follows (Figure 6):

• Reconstructed side: 26.31 ± 1.28 mL.
• **Unaffected side:** 25.71 ± 1.82 mL.

Paired *t*‐test analysis revealed no statistically significant difference between sides (*p* > 0.05).

Enophthalmos is a common concern in patients undergoing orbital surgery. Normal eyeball position is maintained through the balance between orbital volume and intraorbital soft tissues. Disruption of this balance—due to either orbital expansion or loss of orbital contents—can lead to enophthalmos. However, during reconstruction of the orbital floor following tumor resection, periorbital fat and extraocular muscles were preserved, ensuring maintenance of orbital contents.

In this study, orbital contents and extraocular muscles were preserved in all patients. None of the patients complained of preoperative enophthalmos or experienced visual acuity or ocular motility impairment.

**FIGURE 7:**
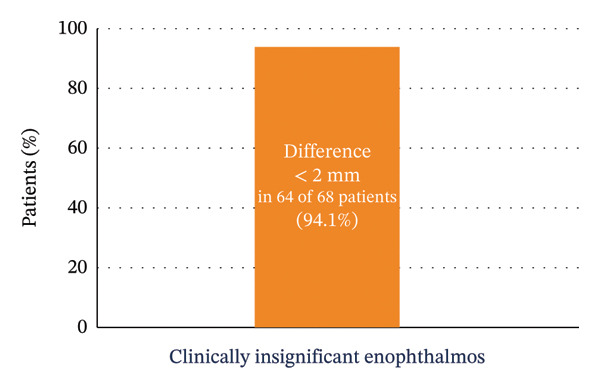
Globe projection difference.

In 64 patients (94.1%), the difference in globe projection between the operated and contralateral sides was less than 2 mm, indicating the absence of clinically significant enophthalmos (Figure 7). Postoperative examinations also showed a low incidence of complications such as diplopia and limited ocular motility.

### 3.2. Functional Outcomes

Postoperatively, 65 of 68 patients (95.6%) demonstrated preserved visual acuity and normal ocular motility. No statistically significant deterioration in visual function was observed (*p* > 0.05) (Table 3).

**TABLE 3:** Postoperative functional outcomes.

Outcome parameter	Result
Preserved visual acuity	65/68 (95.6%)
Transient postoperative diplopia	3/68 (4.4%)
Persistent ocular motility limitation	0 (0%)
Significant orbital volume difference between operated and contralateral sides	None (*p* > 0.05)
Mean orbital volume (operated side)	26.31 ± 1.28 mL
Mean orbital volume (contralateral side)	25.71 ± 1.82 mL
Mean facial symmetry score	8.7 ± 0.9

### 3.3. Complications and Aesthetic Outcomes

**FIGURE 8:**
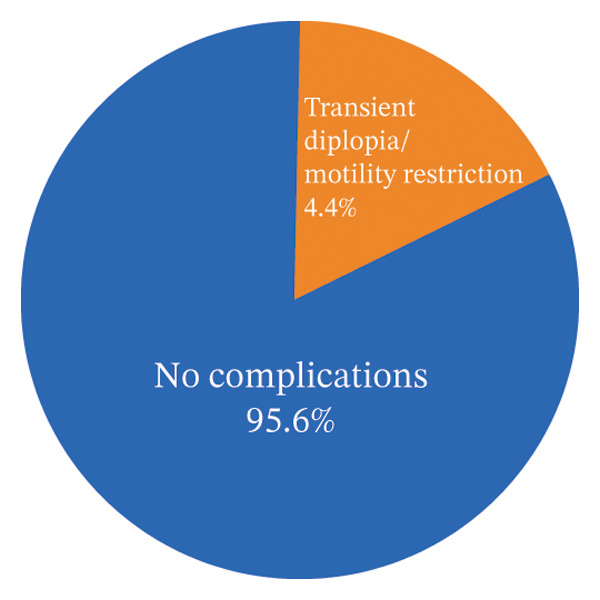
Postoperative complications.

No severe postoperative complications were recorded. Transient diplopia occurred in three patients (4.4%), and temporary limitation of ocular motility was observed in two patients (2.9%). All of these complications resolved during follow‐up (Figure 8).

**FIGURE 9:**
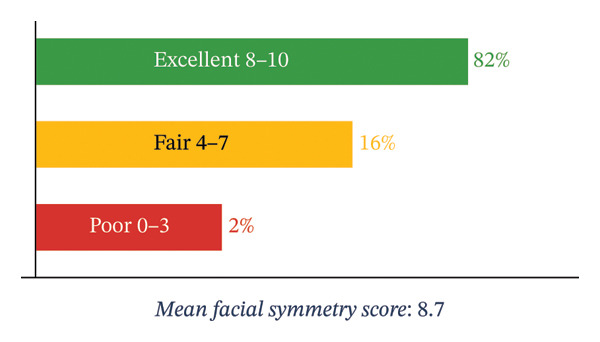
Aesthetic outcomes.

Facial symmetry was evaluated both by patients and surgeons. Patients rated aesthetic outcomes on a scale: excellent (8–10), fair (4–7), or poor (0–3). In this study, aesthetic results were satisfactory in all cases, with all patients expressing satisfaction with postoperative facial symmetry. The mean facial symmetry score as assessed by patients was 8.7 (Figure 9).

## 4. Discussion

Orbital surgery for neoplastic lesions represents a technically demanding field due to the compact anatomical arrangement of critical neurovascular structures within the orbital cavity. The close relationship between the optic nerve, extraocular muscles, lacrimal gland, and orbital walls necessitates a tailored and anatomy‐driven surgical strategy based on tumor size, histology, and precise localization.

Recent studies have emphasized the importance of selecting the orbitotomy approach according to tumor localization within orbital compartments. Lateral orbitotomy remains the preferred technique for lateral intraconal and lacrimal gland tumors, whereas minimally invasive endoscopic endonasal approaches provide improved access to medial and inferior lesions with reduced surgical morbidity.

The lateral orbitotomy technique was first described by Kronlein, who introduced a trans‐temporal approach involving temporary removal of the lateral orbital wall to access retrobulbar lesions [18]. Subsequent refinements, including those proposed by Berke, improved surgical exposure and cosmetic outcomes [19]. With the advancement of endoscopic technology and intraoperative navigation, modern orbitotomy techniques now allow more precise tumor resection with reduced morbidity.

Various orbital approaches are indicated depending on tumor characteristics. Primary orbital tumors such as meningiomas, cavernous hemangiomas, gliomas, neurofibromas, lymphoid tumors, lacrimal gland tumors, and dermoid cysts may require different surgical corridors. Lateral orbitotomy remains particularly effective for lesions involving the lacrimal gland and lateral intraconal or extraconal retrobulbar space [20].

Later, modified methods of classical lateral orbitotomy were proposed, thanks to improved intraoperative technologies and the introduction of endoscopy, which improved cosmetic results [21–25]. However, contraindications include significant intracranial extension and deeply seated medial lesions.

Primary orbital tumors (meningioma, hemangioma, neurofibroma, glioma, lymphoid tumors, and lacrimal gland and dermoid tumors), extraorbital tumors with local extension to the orbital cavity, and secondary tumors extending to the orbital cavity (meningiomas and nasal cavity cancers) are removed through different orbital entrances [26–32].

Tumor size and location are important predictors of surgical complexity and complication risk. Large tumors often benefit from internal debulking prior to peripheral dissection to protect adjacent structures. Lesions located in the inferior orbital compartment present specific technical challenges. In such cases, the inferior orbitotomy or transmaxillary approach described in this study provided safe and adequate access to the retro‐orbital space while preserving orbital integrity.

The choice of surgical approach must therefore be individualized according to tumor size, compartment (intraconal vs. extraconal), and relationship to the optic nerve. Contemporary orbital surgery increasingly emphasizes minimally invasive techniques and anatomical preservation. Recent retrospective analyses of large patient cohorts have confirmed that individualized orbitotomy strategies improve postoperative outcomes and reduce complication rates [33].

In the present series of 68 patients, a differential approach based on tumor localization allowed preservation of visual acuity and ocular motility in the majority of cases.

In our series, a localization‐based strategy allowed appropriate selection among lateral, inferior, supraorbital, and endoscopic endonasal approaches. Lateral orbitotomy proved particularly effective for lateral intraconal and lacrimal gland tumors, providing excellent exposure and favorable cosmetic results. Inferior and transmaxillary approaches offered safe access to retro‐orbital inferior lesions while preserving orbital volume and minimizing postoperative enophthalmos.

Postoperative orbital volume measurements demonstrated no statistically significant differences between reconstructed and unaffected sides, confirming anatomical restoration. The absence of significant enophthalmos and the high mean facial symmetry score further support the functional and aesthetic reliability of the applied techniques.

In recent years, endoscopic endonasal surgery has gained increasing attention as a minimally invasive alternative for selected orbital lesions, particularly those located in the medial intraconal space or orbital apex. Several contemporary clinical series have demonstrated that the endoscopic endonasal approach allows effective tumor removal while minimizing external incisions and soft tissue trauma [34].

Furthermore, case series published between 2021 and 2024 report favorable outcomes with endoscopic endonasal resection of orbital apex tumors, including improved visualization, reduced postoperative morbidity, and satisfactory visual outcomes in carefully selected patients [35].

The integration of computer‐assisted planning and intraoperative navigation represents a significant advancement in orbital surgery. Three‐dimensional reconstruction based on mirror imaging of the contralateral orbit enhances anatomical accuracy and reduces intraoperative uncertainty. Image‐guided surgery improves orientation within the confined orbital space and helps minimize the risk of injury to critical neurovascular structures.

Endoscopic endonasal approaches, particularly for medial and inferior lesions, further minimize tissue disruption and improve cosmetic outcomes without compromising surgical radicality. In addition, multidisciplinary collaboration among ophthalmologists, neurosurgeons, otolaryngologists, radiologists, and pathologists is essential to optimize diagnostic accuracy and therapeutic outcomes.

Overall, our results support a differential, anatomy‐driven strategy in orbital tumor management, emphasizing preservation of orbital volume and critical structures to optimize long‐term functional outcomes. The application of modern surgical technologies, including endoscopy and navigation systems, further enhances surgical precision and safety. These developments have significantly expanded the surgical armamentarium available for the management of orbital tumors.

### 4.1. Limitations

This study has several limitations. First, its retrospective design may introduce selection bias. Second, the sample size, although clinically meaningful, limits subgroup statistical comparison between different surgical approaches. Third, long‐term oncological outcomes were not analyzed separately for benign and malignant tumors. Finally, quality‐of‐life assessment relied primarily on subjective cosmetic scoring rather than validated patient‐reported outcome measures.

Future prospective, multicenter studies with standardized functional and aesthetic outcome metrics are warranted to further validate these findings.

## 5. Conclusion

Orbitotomy techniques tailored to tumor localization provide safe and effective management of orbital neoplasms. In this clinical series, visual acuity and ocular motility were preserved in most patients, and postoperative orbital symmetry was maintained. Lateral orbitotomy remains a reliable method for large and deep lateral lesions, while minimally invasive endoscopic approaches with intraoperative navigation offer durable functional and aesthetic outcomes with low complication rates.

## Funding

This work was not funded.

## Ethics Statement

The study was reviewed and approved by the Ethics Committee of Yerevan State Medical University (IRB no. 28) and was conducted in accordance with the ethical principles of the World Medical Association Declaration of Helsinki.

## Consent

Written informed consent was obtained from all participants and/or their legal guardians for publication of the case reports, accompanying images, and the data included in this manuscript.

## Conflicts of Interest

The authors declare no conflicts of interest.

## Data Availability

Data sharing is not applicable to this article as no datasets were generated or analyzed during the current study.
